# High virologic response rate after second-line boosted protease inhibitor-based antiretroviral therapy regimens in children from a resource limited setting

**DOI:** 10.1186/1742-6405-9-20

**Published:** 2012-06-18

**Authors:** Thanyawee Puthanakit, Gonzague Jourdain, Piyarat Suntarattiwong, Kulkanya Chokephaibulkit, Umaporn Siangphoe, Tulathip Suwanlerk, Wasana Prasitsuebsai, Virat Sirisanthana, Pope Kosalaraksa, Witaya Petdachai, Rawiwan Hansudewechakul, Naris Waranawat, Jintanat Ananworanich

**Affiliations:** 1 The HIV Netherlands Australia Thailand Research Collaboration (HIV-NAT), Bangkok, Thailand; 2 Department of Pediatrics, Faculty of Medicine, Chulalongkorn University, Bangkok, Thailand; 3 Institut de Recherche pour le Développement (IRD), UMI 174, Program for HIV Prevention and Treatment, Department of Medical Technology, Faculty of Associated Medical Sciences, Chiang Mai University, Chiang Mai, Thailand; 4 Queen Sirikit National Institute of Child Health, Bangkok, Thailand; 5 Department of Pediatrics, Faculty of Medicine Siriraj Hospital, Mahidol University, Bangkok, Thailand; 6 Research Institute for Health Sciences, Chiang Mai University, Chiang Mai, Thailand; 7 Department of Pediatrics, Faculty of Medicine, Khon Kaen University, Khon Kaen, Thailand; 8 Petchburi Hospital, Petchburi, Thailand; 9 Chiang Rai Regional Hospital, Chiang Rai, Thailand; 10 South East Asia Research Collaboration with Hawaii, Hawaii, Thailand; 11 Department of Medicine, Faculty of Medicine, Chulalongkorn University, Bangkok, Thailand

**Keywords:** Pediatric HIV, Drug resistance, Second-line antiretroviral therapy, Protease-inhibitors, Resource limited settings

## Abstract

**Background:**

Limited data exist for the efficacy of second-line antiretroviral therapy among children in resource limited settings. We assessed the virologic response to protease inhibitor-based ART after failing first-line non-nucleoside reverse transcriptase inhibitor (NNRTI)-based regimens.

**Methods:**

A retrospective chart review was conducted at 8 Thai sites of children who switched to PI –based regimens due to failure of NNRTI –based regimens. Primary endpoints were HIV RNA < 400 copies/ml and CD4 change over 48 weeks.

**Results:**

Data from 241 children with median baseline values before starting PI-based regimens of 9.1 years for age, 10% for CD4%, and 4.8 log_10_ copies/ml for HIV RNA were included; 104 (41%) received a single ritonavir-boosted PI (sbPI) with 2 NRTIs and 137 (59%) received double-boosted PI (dbPI) with/without NRTIs based on physician discretion. SbPI children had higher baseline CD4 (17% vs. 6%, p < 0.001), lower HIV RNA (4.5 vs. 4.9 log_10_ copies/ml, p < 0.001), and less frequent high grade multi-NRTI resistance (12.4% vs 60.5%, p < 0.001) than the dbPI children. At week 48, 81% had HIV RNA < 400 copies/ml (sbPI 83.1% vs. dbPI 79.8%, p = 0.61) with a median CD4 rise of 9% (+7%vs. + 10%, p < 0.005). However, only 63% had HIV RNA < 50 copies/ml, with better viral suppression seen in sbPI (76.6% vs. 51.4%, p 0.002).

**Conclusion:**

Second-line PI therapy was effective for children failing first line NNRTI in a resource-limited setting. DbPI were used in patients with extensive drug resistance due to limited treatment options. Better access to antiretroviral drugs is needed.

## Introduction

The most commonly used first-line antiretroviral therapy (ART) in HIV-infected children in resource-limited settings (RLS) is a non nucleoside reverse transcriptase inhibitor (NNRTI)-based treatment [1,2]. Data from individual cohorts in Thailand [3], Uganda [4] Cambodia[5] and those from a meta-analysis of 1457 children [1] showed that 70–81% had viral suppression after 1 year of first-line treatment. Several pediatric programs in RLS have begun to provide second-line therapy; 5.8% and 20% among cohorts of HIV-infected children in South Africa [6] and the TREAT Asia regional network [7], but treatment outcomes data are limited. The second-line regimen for children failing NNRTI-based treatment in all treatment guidelines is a low-dose ritonavir boosting protease inhibitors (boosted PI) in combination with 2 nucleoside reverse transcriptase inhibitors (NRTIs) [8,9] which was reasonably effective [10].

However, in RLS without routine virologic monitoring, the diagnosis of treatment failure is usually late, of which brings about concerns for multi-NRTI resistance [11]. Reported incidence rate of multi-NRTI drug resistance from cohort with routine viral load monitoring is only 8% [12] compared to 36% seen in settings in which treatment failure was detected by clinical or immunologic criteria [13]. In such cases in RLS, the only effective anti-retroviral (ARV) drug option available for second-line therapy is boosted PI with limited choices for effective NRTIs. Often times, double-boosted PI regimens are used to provide 2 active agents in the regimen. Double-boosted PIs is an alternative second-line therapy option in the Thai National Guideline; however, there have been limited data of its safety and efficacy in HIV-infected children [14].

The primary objective of this study is to describe the immunologic and virologic efficacy of second-line ARV regimens containing either single- or double-boosted PI over a 48-week period in children with NNRTI-based treatment failure in Thailand.

## Material and methods

### Study design and subjects

We formed a network of 8 large pediatric HIV centers in Thailand to retrospectively collect treatment outcome data of all children who failed NNRTI-based therapy and received ritonavir-boosted PI regimen as a second-line drug regimen. Immunologic treatment failure was defined according to the Thai guideline either CD4 percentage decline > 5 percentage point in a patient with CD4% less than 15, or CD4 cell count drop > 30% of baseline within 6 months [15]. Before 2006, the access to plasma HIV RNA monitoring was limited and it was performed after immunologic failure was suspected. Virological failure was defined as HIV RNA > 1000 copies/ml after at least 6 months of antiretroviral therapy. After 2006, annual HIV RNA was accessible through the national program. In some cases, genotypic resistance testing was performed before switching to second-line regimens, and the test was performed only if plasma HIV RNA was > 1,000 copies/ml.

Cases selected were HIV-infected children aged < 18 years, with a documented history of immunologic or virologic failure on NNRTI-based ART who received ritonavir-boosted PIs-based regimen for at least 24 weeks. They were excluded if they had previously received PIs treatment prior to the ritonavir-boosted PIs for longer than 30 days, or received second-line drugs not belonging to the NRTI, NNRTI, and PI classes. Standardized forms were used for retrospective hospital chart extraction: include demographics, CDC HIV clinical classification, history of ART, CD4 cell count and percentage, and genotypic resistance test result before switching to PI-based HAART. Follow up CD4, plasma HIV RNA and adverse events after switch to PI regimen were extracted and censored at last patient visit. After switching to second-line therapy, CD4 was uniformly monitored every 6 months and HIV RNA every 6–12 months. Single-boosted PI was defined as low dose ritonavir combined with one other PI drug. Double-boosted PI was defined as low dose ritonavir combined with another two PI drugs, or therapeutic dose ritonavir (350–400 mg/m^2^/dose) combined with one other PI drug.

The HIV RNA testing was performed using 50 copies/ml as the limit of detection, except for some cases in the early 2000s which used 400 copies/ml as the detection limit. Genotypic resistance testing was performed by TrueGene HIV-1 Genotyping system (Visible Genetics, Inc., Toronto, Canada) at 5 sites, ViroSeq HIV-1 Genotyping System (Celera Diagnostics, Alameda, Calif.) at 1 site and with an in house method using Stanford and International AIDS society (IAS) database [16] at 2 sites. The study was approved by the Ethics Committees at all sites.

### Statistical analysis

Primary endpoints were the proportion of children with plasma HIV RNA < 400 copies/ml at week 48 and the CD4 changes at 48 weeks after switching to second-line PI therapy. Secondary endpoints were the proportion of children with plasma HIV RNA < 50 copies/ml and the prevalence of adverse events. The analysis was performed using available data.

The cumulative probability of virologic treatment failure defined as HIV RNA > 400 copies/ml after at least 6 months of second-line boosted PI regimen was calculated using the Kaplan Meier estimates. The data included patients who had HIV RNA result at least once after 24 weeks of treatment and censored at 24 months or earlier in cases of discontinuation of the follow-up. The difference between single- and double -boosted PI was tested using the log rank test.

The predictors for treatment failure defined as HIV RNA < 400 copies/ml at week 48 were explored in a logistic regression model. Factors included CD4 cell count at time of switch to boosted PI regimen (dichotomized as > 100 cells/mm^3^), plasma HIV RNA at time of switch to boosted PI regimen (dichotomized as > 10,000 copies/ml), ART regimen as single-boosted PI or double-boosted PI, use of lopinavir/ritonavir (LPV/r) –containing regimen, and grade of resistance mutations. Definition of high grade multi-NRTI resistance was defined as ≥ 4 thymidine analog mutations (TAMs) or the presence of Q151M or 69 insertion. Low grade multi-NRTI resistance was defined as 1 to 3 TAMs. Factors with p-value < 0.2 in the univariate analysis were tested in the multivariate analysis. Analyses were performed using SAS version 9.1(SAS Institute, Cary, NC, USA).

## Results

### Patient characteristics and antiretroviral regimen

Between September 2002 and June 2007, 241 children were enrolled, including 1, 11, 62, 63, 60, and 44 children per calendar year. There were 104 children who switched to single-boosted PI and 137 children who switched to double-boosted PI (137 children) at 8 HIV pediatric clinics. The baseline characteristics of the children are shown in Table 1. The medians (interquartile range [IQR]) were 9.1 (7.2-11.1) years for age and 2.2 (1.5-2.9) years for duration of NNRTI-based regimen. Among 203 children who had genotypic resistance testing performed, 195 children (96%) had the test result prior to switching to PI-based regimen and the remaining had the test done on stored samples at a later date. The ARV regimens were chosen by physician’s discretion based on ARV treatment history, genotypic resistance pattern (if available), and the availability of ARV drugs. Indinavir has been available in national program, since 2002, while LPV/r has been available in national antiretroviral program since 2005. The median follow-up time was 26.0 months (IQR 12.7-36.8). Overall, at the time of switch to second line regimens, the children who were prescribed single-boosted PI were younger (median: 8.9 versus 9.4 years, P = 0.046), had a significantly higher baseline CD4% (17% versus 6%, p < 0.001), a lower baseline plasma HIV RNA (4.5 log_10_ versus 4.9 log_10_ copies/ml; p <0.001), and a lower proportion of genotypes with multi-NRTI resistance mutation (12.4% versus 60.5%, p < 0.001) compared with children who received double-boosted PI regimen.

**Table 1:** Baseline Characteristics among 241 HIV-infected children who received second-line protease inhibitor –based antiretroviral therapy

**Characteristics**	**All (n = 241)**	**Single-boosted PIRegimens**^**1**^**(n = 104)**	**Double-boosted PI regimens**^**1**^**(n = 137**)	**P-value**
Gender: Male	117 (49)	55 (53)	62 (45)	0.24
Age, years	9.1 (7.2-11.1)	8.9 (6.1-11.1)	9.4 (7.6-11.2)	**0.046**
**Prior to initiate NNRTI regimen**				
CDC clinical staging				**0.04**
N	10 (4.2)	7 (6.7)	3 (2.2)	
A	58 (24.3)	29 (27.9)	29 (21.6)	
B	113 (47.3)	39 (37.5)	74 (54.8)	
C	58 (24.3)	29 (27.9)	29 (21.5)	
CD4 percentage	5 (1–10)	3.5 (1–11)	5 (2–10)	0.53
CD4 cell/mm^3^	109 (27–410)	109 (21–395)	112 (28–413)	0.51
**Prior to switch to second-line boosted -PI regimen**				
Weight for age Z-score	−1.7 (−2.1 to −0.9)	−1.4 (−1.9 to −0.7)	−1.9 (−2.3 to −1.4)	**<0.001**
CD4% (n = 239)	10 (4–18)	17 (7–24)	6 (2–12)	**<0.001**
CD4 cell/mm^3^ (n = 238)	195 (70–442)	379 (165–659)	123 (38–273)	**<0.001**
HIV RNA, log _10_ copies/mL (n = 227)	4.8 (4.3-5.3)	4.5 (3.9-5.1)	4.9 (4.5-5.4)	**<0.001**
**Antiretroviral treatment history**				
Mono/dual NRTI exposure prior to NNRTI-based regimen	71 (29.5)	23 (22.1)	48 (35.0)	**0.03**
Duration on NNRTI-based treatment, years	2.2 (1.5-2.9)	2.3 (1.5-3.1)	2.0 (1.4-2.9)	**0.04**
NNRTI regimen				**0.01**
Nevirapine	167 (69.3)	81 (77.9)	86 (62.8)	
Efavirenz	74 (30.7)	23 (22.1)	51 (37.2)	
NRTI-backbone				
d4T/3TC	142(58.9)	71 (68.2)	71 (51.8)	**0.015**
AZT/3TC	60 (24.9)	25(24.0)	35(25.6)	
d4T/ddI	17 (7.1)	4 (3.9)	13(9.5)	
AZT/ddI	14(5.8)	3(2.9)	11(8.0)	
ddI/3TC	8 (3.3)	1 (1.0)	7 (5.1)	
Multi-NRTI resistance^2^				**<0.001**
No TAMs	45/203(22.2)	38/89 (42.7)	7/114 (6.1)	
Low grade multi-NRTI	78/203 (38.4)	40/89 (44.9)	38/114 (33.3)	
High grade multi-NRTI	80/203 (39.4)	11/89 (12.4)	69/114 (60.5)	

Data are presented in median (IQR) or number (%).

^1^Single boosted PI regimens include ritonavir-boosted Protease inhibitor plus 2 nucleoside reverse transcriptase. Double-boosted PI regimens included 2 protease inhibitors plus ritonavir with/without NRTI/NNRTI.

^2^Definition of multi NRTI resistance: high grade is defined as ≥ 4 thymidine analog associated mutations (TAMs) or the presence of Q151M or 69Insertion. Low grade multi NRTI resistance is defined as 1–3 TAMs and without Q151M and 69insertion.

The single-boosted PI regimens included 55 (53%) indinavir/r (IDV/r) and 49 (47%) lopinavir/r (LPV/r). The NRTI backbones used were 50 (48%) zidovudine (AZT) plus lamivudine (3TC), 29 (28%) AZT plus didanosine (ddI), 17 (16%) ddI plus 3TC and 8 (8%) miscellaneous. Among children in the double -boosted PI group, 77 (56%) did not receive any NRTI, 37 (27%) received 3TC as the only NRTI, and 23 (17%) received other NRTIs. The double-boosted PI regimens were LPV/r + IDV in 46 (34%) children, IDV/r + Nelfinavir (NFV) in 24 (18%), LPV/r + Saquinavir (SQV) in 20 (15%), LPV/r + NFV in 17(12%), IDV + RTV in 16(12%), IDV/r + SQV in 8 (6%) and other PIs in 6 (4%) children.

### Efficacy of second-line boosted protease inhibitor regimens

At week 24, 223 had CD4 information and 190 had plasma HIV RNA information. The overall median (IQR) CD4% change was 5 (1–8) % with more CD4 rise in children who received double- compared to single-boosted PI (+5% vs. +4%, p < 0.016). Overall 78% achieved HIV RNA < 400 copies/ml. The proportion with HIV RNA < 400 copies/ml was not different between groups; 80.9% (72/89) in single- vs. 76.2% (77/101) in double-boosted PI regimens. The proportion with HIV RNA below 50 copies/ml was 59% overall with a higher proportion in single- compared to double-boosted PI groups: 66.7% (58/87) vs. 51.2% (44/86) p = 0.038).

The outcomes at week 48 after initiation of boosted PI regimens are shown in Table 2. The increase in CD4 percentage was significantly higher among children who received double-boosted PI. Overall, 81.3% had HIV RNA < 400 copies/ml (single-versus double-boosted PI: 83.1% vs. 79.8%, p = 0.61). Sixty-three percent had plasma HIV RNA < 50 copies/ml; a higher proportion of these were children who received single-boosted PI compared with those receiving double-boosted PI (76.6% vs. 51.4%, p = 0.002).

**Table 2:** Outcomes after 48 weeks of boosted protease inhibitor regimens

**Outcomes**	**Week 48**			
	**Total**	**Single –boosted PI**	**Double-boosted PI**	**P-value**
Median (IQR) CD4%	20 (15–26) (n = 200)	24 (18–30) (n = 83)	17 (14–22) (n = 117)	**< 0.001**
Median(IQR) CD4% gain	10(1–7) (n = 199)	7(2–12) (n = 83)	10(6–15) (n = 116)	0.002
% with HIV RNA < 400 copies/ml	81.3 (n = 144)	83.1 (n = 65)	79.8 (n = 79)	0.610
% with HIV RNA < 50 copies/ml	63.0 (n = 138)	76.6 (n = 64)	51.4 (n = 74)	**0.002**
% with Cholesterol > 200 mg/dl	42.5 (n = 162)	34.3 (n = 70)	48.9 (n = 92)	0.062
% with Triglyceride > 150 mg/dl	62.4 (n = 157)	61.8 (n = 68)	62.9 (n = 89)	0.882
% with Low density lipoprotein > 130 mg/dl	31.8 (n = 88)	16.2 (n = 37)	43.1 (n = 51)	**0.007**

Subgroup analysis was performed for the outcome HIV RNA < 400 copies/ml at week 48. Among children in single-boosted PI group, 30/36 (83.3%) of children who received indinavir/r and 24/29 (82.8%) of children who received lopinavir/r had undetectable viral load (P-value 0.655). Among children in double boosted PI group, 47/57 (82.5%) of children who did not receive any NRTI and 16/22 (72.7%) of children who received NRTIs had undetectable viral load (p-value 0.252).

### Adverse events

During the follow-up period, there were 31 adverse events that led to discontinuation or substitution of PI drugs. The median time of discontinuation or substitution were 13.3 months (IQR 7.1-26.0) after start PI-based regimen. There were 20 events for IDV-related toxicity (13% of children who used IDV)due to nephrotoxicity (n = 11), hyperbilirubinemia (n = 4), nausea vomiting (n = 2), intolerance (n = 1), blurred vision (n = 1), hypertriglyceridemia (n = 1); 5 events for full dose RTV (31% of children who used full dose of RTV) due to intolerance (n = 3), hyperbilirubinemia (n = 1) and hypertriglyceride (n = 1); 4 events for NFV (10% of children who used NFV) due to intolerance (n = 2), rash (n = 1) and diarrhea (n = 1); 1 event for SQV (4% of children who used SQV) due to intolerance and 1 event for LPV/r (0.8% of children who used LPV/r) due to dyslipidemia. Proportion of children with elevated low density lipoprotein was significantly more in the double-boosted compared to single-boosted PI groups (Table 2).

### Cumulative probability of virologic treatment failure

**Figure 1:**
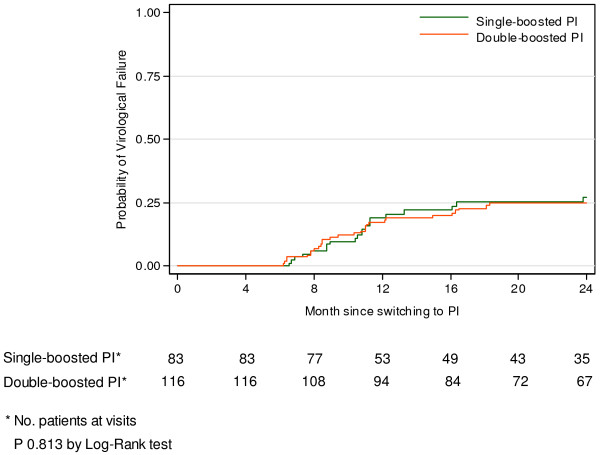
Kaplan Meier estimated for time to treatment failure, defined as HIV RNA > 400 copies/ml, after switching to second line PI regimen.

The cumulative probability of virologic treatment failure was analyzed by including data from 199 patients who had HIV RNA results at least once after 6 months of boosted PI regimens. The Kaplan Meier estimate is shown in Figure 1. The cumulative probabilities of having HIV RNA > 400 copies/ml at 24 months of treatment were 0.28 (95% CI 0.19-0.40) for children who received single-boosted PI and 0. 27 (95% CI 0.18-0.39) for children on double-boosted PI, p = 0.813 by log rank test.

### Predictors for virologic suppression at week 48 after boosted PI regimen

The predictors for virologic suppression at week 48 were explored by multivariate logistic regression analysis as shown in Table 3. Male, young age and high weight for age z-score were significantly associated with virologic suppression. Male had a 2.9 times odds of achieving viral suppression compared to female. Chances for viral suppression were more likely in children younger than 9 years (3.6 times odds) and those with weight for age z-score > −1.7 (4.2 times odds). There was a trend for a better virologic suppression rate among children who switched to boosted PI regimen when their CD4 count was > 100 cell/mm^3^ or plasma HIV RNA levels < 4 log_10_ copies/ml but these did not reach statistical significance (p > 0.05).

**Table 3:** The predictors for virological suppression defined as HIV viral load < 400 copies/ml at week 48 of boosted protease inhibitor-based antiretroviral regimens

	**Proportion of children with viral suppression n(%)**	**Univariate analysis**		**Multivariate analysis**	
		**Odd Ratios (95%CI)**	**P-value**	**Odd Ratios (95%CI)**	**P-value**
ARV regimens					
Single-boosted PI	54/65(83.1%)	1		1	
Double-boosted PI	63/79 (79.8%)	0.80 (0.34-1.88)	0.611	0.82 (0.27-2.42)	0.712
Gender					
Male	62/69 (89.9%)	3.22 (1.27-8.20)	0.014	2.85 (1.06-7.66)	0.018
Female	55/75 (73.3%)	1		1	
Age, years					
< 9.1	67/76 (88.2%)	2.68 (1.12-6.46)	0.028	3.60 (1.25-10.36)	0.038
≥ 9.1	50/68 (73.5%)	1		1	
Weight for age Z-score					
< −1.7	42/59 (71.2%)	1		1	
≥ −1.7	64/72 (88.9%)	3.24 (1.28-8.18)	0.013	2.42 (0.85-6.84)	0.097
CD4 at time of switch to second line regimen					
< 100 cell/mm3	30/42 (71.4%)	1		1	
≥100 cell/mm3	84/99 (84.9%)	2.24 (0.94-5.33)	0.068	2.12 (0.71-6.36)	0.178
Plasma HIV RNA at time of switch					
< 4 log_10_ copies/ml	25/29 (86.2%)	1.53 (0.48-4.86)		-	-
≥ 4 log_10_ copies/ml	25/111 (80.4%)	1	0.474		
Mono/dual NRTI exposure					
Yes	41/50 (82.0%)	1.08 (0.45-2.62)	0.867		
No	76/94 (80.9%)	1			
Duration on NNRTI-based					
< 2.2 year	69/84(82.1%)	1.15 (0.50-2.67)	0.745		
≤ 2.2 year	48/60(80.0%)	1			
NNRTI –based regimen					
NVP-based	81/96 (84.4%)	1.80 (0.77-4.23)	0.178	1.24 (0.43-3.55)	0.691
EFV-based	36/48 (75.0%)	1		1	
Multi-NRTI resistance			0.809	-	-
No TAMs	25/32 (78.1%)	1			
Low grade multi-NRTI	35/44 (79.6%)	1.09 (0.36-3.31)			
High grade multi-NRTI	36/43 (83.7%)	1.44 (0.45-4.62)			
LPV/r in the regimen					
Yes	60/71 (84.5%)	1.53 (0.66-3.58)	0.325		
No	57/73 (78.1%)	1			

## Discussion

Our study provides information on outcomes of second-line ART in NNRTI-failing children as part of a large multicenter observational study in a RLS. This study showed good virologic efficacy of second-line boosted PI regimens with 81% achieving HIV RNA < 400 copies/ml at 48 weeks of treatment. Children with more advanced HIV disease were preferentially treated with double-boosted PI versus single-boosted PI regimens. Both regimen types performed equally well in suppressing HIV below 400 copies/ml but further suppression to less than 50 copies/ml was significantly less with double-boosted PI.

This study supports recycling of 2NRTIs in combination with a potent boosted PI as second-line therapy in children. The 2010 WHO guideline recommend replacing AZT/3TC or d4T/3TC in the first line regimen with abacavir (ABC)/3TC or ABC/ddI [17]; however, that practice is rare in Thailand due to the high cost of ABC rendering it unavailable in the Thai national ARV program. Therefore, recycling of inactive or partially active 2NRTIs such as AZT/3TC, AZT/ddI or ddI/3TC was common in this study. At the time of the study, tenofovir (TDF) was not yet approved for adolescents nor was it available in Thailand. Similarly, LPV/r is the PI of choice in the WHO guideline but it was in limited supply in Thailand before 2005, resulting in the use of a more toxic drug, IDV, in half of our children.

The virologic response to second-line single boosted PI regimen in our study compares favorably to reports from other countries [15-17]. In a French cohort, 92% on LPV/r second line regimens had HIV RNA < 400 copies/ml despite having lower CD4 (14.8%) and higher HIV RNA (4.8 log_10_ copies/ml) than our children. The Spanish multicenter retrospective observational study showed 71.5% of PI-experienced children on LPV/r-based having HIV RNA < 400 copies/ml [10]. A long term cohort of children treated with LPV/r-based regimen showed that 81% remained on therapy after more than 4 years; 75% of those children had HIV RNA < 400 copies/ml at their last visit. [18] In another pediatric study, patients tended to have better virologic outcome if they were on LPV/r as supposed to unboosted PI, nelfinavir [19]. We also saw a trend towards better virologic outcome among children who received LPV/r, but this did not reach statistical significance. In Malawi, 10% of adults died after initiating second-line regimen and 85% of the survivors had viral suppression at 12 months [20].

Double-boosted PI is an acceptable alternative second-line therapy option in Thailand for children with late treatment failure who have few or no fully active drugs aside from PIs [15]. The pediatric HIV-NAT 017 study which treated children with second-line SQV and LPV/r reported a viral suppression rate similar to that observed in our study with 64% having HIV RNA < 50 copies/ml at week 48 [21]. A non-randomized study in Thai adults showed that the double-boosted PI regimen was not as potent in suppressing HIV RNA to below 50 copies/ml when compared to the single-boosted PI regimen among patients with low grade multi- NRTI resistance mutations [22]. Of note is the inability of that study to compare the two regimen types among patients with high grade multi-NRTI resistance mutations. Our study showed that double-boosted PI had a higher rate of low level viremia, HIV RNA between 50 and 400 copies/ml, compared to single-boosted PI. This could possibly be confounded by indication that children with more advanced disease received double-boosted PI regimen or due to inferior potency of a PI mono-class regimen. A recent randomized study of second-line PIs in Thai adults showed mono LPV/r treatment to result in significantly more low level viremia than a 3-drug regimen with TDF, 3TC and LPV/r [23]. Another second-line study in Thai adults who received a single active drug, LPV/r, together with 3TC to reduce viral fitness also showed that only 67% achieved HIV RNA <50 copies/ml with 16% having low viremia between 50–400 copies/ml [24]. The recent review literature [25] and meta-analysis [26]on protease inhibitor monotherapy also showed a slightly inferior virological efficacy of protease inhibitor monotherapy than that of protease inhibitor plus nucleosides. However, failure of protease inhibitor monotherapy does not imply losing therapeutic options, usually reintroduction of nucleosides can lead to virologic suppression. [25,26] The higher rates of dyslipidemia with double-boosted PI and the higher pill burden further limits its use particularly if newer and more potent ARVs with favorable lipid profile e.g. atazanavir, darunavir, and raltegravir becomes available to children in RLS.

There is a lack of evidence to inform the optimum time to switch to second line regimens in RLS. The WHO guideline, relying heavily on a public health approach to care, recommends switching to second-line regimen when CD4 is < 100 cells/mm^3^ or HIV RNA is > 5,000 copies/ml [27]. We demonstrated a trend towards better virologic suppression following second-line therapy in children who switched when their CD4 was > 100 cell/mm^3^ or HIV RNA was < 10,000 copies/ml. Delayed in switching to second-line therapy may lead to resistance mutations accumulation which would be problematic in RLS where ARV options are limited. The PENPACT-1 study showed that children failing first-line NNRTI who were randomized to a delayed switch at HIV RNA > 30,000 copies/ml had more TAMs compared to those who switched at HIV RNA > 1,000 copies/ml [28] This however did not affect the overall virologic outcome after second-line therapy in that study likely due to several reasons: children who failed had low rates of NRTI resistance of 1-5%, they had no CD4 failure, and they had access to routine HIV RNA monitoring, genotyping and good ARV options. The situation in RLS may be quite different as switching occurs later with immunologic or clinical failure coupled with the lack of HIV RNA and genotyping monitoring, and limited ARV options.

This study has several limitations. First is incomplete data, which is an inherent limitation of a retrospective study design. The information of adherence to treatment is not captured. Second, the lack of randomization of single- versus double-boosted PI regimens hinders the ability to directly compare the outcome between these two regimens. Third, the duration of NNRTI-based first line regimen in our study was relatively short, around 2 years. Furthermore, we have access to laboratory monitoring including CD4, plasma HIV RNA and genotypic resistance testing to guide the design of the new regimens which may limit the applicability of our results to other resource-limtted settings where treatment failure is detected later and genotyping is unavailable. However, the constraint in available ARV choices in our population prohibited us to fully utilize genotyping information. Therefore, we believe that our data represent typical clinical care settings in most resource-limited countries.

In conclusion, this study provides security to clinicians in RLS to give children second-line treatment despite drug choice and laboratory monitoring constraints. Although we saw a good success rate in our study, one-fifth did not achieve viral suppression. Such children in RLS currently have no treatment option beyond second-line regimens. This emphasizes the urgent need to derive strategies to study, plan and procure new drugs and drug classes for children with treatment failure worldwide.

### The HIV-NAT 086 study team

The HIV Netherlands Australia Thailand Research Collaboration (HIV-NAT), The Thai Red Cross AIDS Research Centre, Bangkok: T. Bunupuradah, C. Phasomsap, P. Kaew-on.

Institut de Recherche pour le Développement (IRD), UMI 174, Program for HIV Prevention and Treatment, Department of Medical Technology, Faculty of Associated Medical Sciences, Chiang Mai University, Chiang Mai, Thailand, The Global Fund to Fight AIDS, Malaria and Tuberculosis supported drug and laboratory monitoring for some children: S Kanjanavanit, Nakornping Hospital, Somdej Pranangchao Sirikit Hospital, Chonburi, Somdej Pranangchao Sirikit Hospital, Chonburi: T Hinjiranandana, Bhumibol Adulyadej Hospital, Bangkok: P Layangool, Somdej Prapinklao Hospital, Bangkok: N Kamonpakorn, Mae Chan Hospital, Chiang Rai: S Buranabanjasatean, Prapokklao Provincial Hospital, Chantaburi: C Ngampiyaskul.

Queen Sirikit National Institute of Child Health, Bangkok: T. Chotpitayasunondh, S. Chanpradub, P. Leawsrisuk. Department of Pediatrics, Faculty of Medicine, Siriraj Hospital, Mahidol University: S. Chearskul, N. Vanprapar, W. Phongsamart, K. Lapphra, P. Chearskul, O. Wittawatmongkol, W. Prasitsuebsai, K. Intalapaporn, N. Kongstan, N. Pannin, A. Maleesatharn, B. Khumcha.

Research Institute for Health Sciences (RIHES), Chiang Mai University, Chiang Mai: L. Aurpibul, N. Wongnum, R. Nadsasarn.

Department of Pediatrics, Faculty of Medicine, Khon Kaen University: P. Lumbiganon, P. Tharnprisan, T. Udompanich. Petchburi Hospital, Petchburi: M. Yentang.

Chiang Rai Regional Hospital, Chiang Rai: A. Khonponoi, N. Maneerat, S. Denjunta, S. Watanaporn, C. Yodsuwan, W. Srisuk, S. Somsri, K. Surapanichadul

## Competing interests

All authors declare no conflict of interest and that member of their immediate families do not have a financial interest in or arrangement with any commercial organization that may have a direct interest in the subject matter of this article.

## Authors’s contribution

TP, GJ, KC, and JA designed the study, collected data, wrote the first draft, reviewed manuscript before submission. PS, TS,WP,VS, PK,WP,RH, NW collected data, reviewed and commented of manuscript before submission. US analyzed, reviewed and commented draft of manuscript before submission. All authors have read and approved the final manuscript.
